# Mitigating the Drawbacks of the L_0_ Norm and the Total Variation Norm

**DOI:** 10.3390/axioms14080605

**Published:** 2025-08-04

**Authors:** Gengsheng L. Zeng

**Affiliations:** Department of Computer Science, Utah Valley University, Orem, UT 84058, USA

**Keywords:** image reconstruction, total variation prior, piecewise constant, limited angle tomography, *L*_0_ norm, 49N30, 49Q05, 65K10, 68U10, 68W20, 68W25, 68W40, 90C23

## Abstract

In compressed sensing, it is believed that the $L_{0}$ norm minimization is the best way to enforce a sparse solution. However, the $L_{0}$ norm is difficult to implement in a gradient-based iterative image reconstruction algorithm. The total variation (TV) norm minimization is considered a proper substitute for the $L_{0}$ norm minimization. This paper points out that the TV norm is not powerful enough to enforce a piecewise-constant image. This paper uses the limited-angle tomography to illustrate the possibility of using the $L_{0}$ norm to encourage a piecewise-constant image. However, one of the drawbacks of the $L_{0}$ norm is that its derivative is zero almost everywhere, making a gradient-based algorithm useless. Our novel idea is to replace the zero value of the $L_{0}$ norm derivative with a zero-mean random variable. Computer simulations show that the proposed $L_{0}$ norm minimization outperforms the TV minimization. The novelty of this paper is the introduction of some randomness in the gradient of the objective function when the gradient is zero. The quantitative evaluations indicate the improvements of the proposed method in terms of the structural similarity (SSIM) and the peak signal-to-noise ratio (PSNR).

## 1. Introduction

The focus of this paper is on the $L_{0}$ norm minimization. If *x* is a scalar, the $L_{0}$ norm of *x* is defined as [1–3].


(1) $${∥x∥}_{L_{0}}=\left\{\begin{array}{lll} 0 & \mathrm{if} & x=0,\\ 1 & \mathrm{if} & x\neq 0. \end{array}\right.$$

If *X* is a vector or a matrix, the $L_{0}$ norm of *X* is defined as

(2) $${∥X∥}_{L_{0}}=\text{The total count of the nonzero elements in}\;X$$

Here we use the term ‘norm’ even though the $L_{0}$ norm is not a true norm in the mathematical sense. It is better to refer to the $L_{0}$ norm as the cardinality function or sparsity measure. A norm must have the positive homogeneity property

(3) ∥kx∥=k∥x∥,

for all *x* in its domain and k>0. Clearly, the $L_{0}$ norm does not satisfy the positive homogeneity property (3). In fact, according to (1), we have

(4) $$∥kx{∥}_{L_{0}}={∥x∥}_{L_{0}},$$

for all *x* in its domain and k>0.

One important application of the $L_{0}$ norm is in compressed sensing, which was introduced in the first decade of this century [4–10]. The principle of compressed sensing is to exactly reconstruct a signal using a small number of samples if the signal is sparse. The problem can be described as a system of linear equations:

(5) AX=P,

where *A* is the system matrix, *P* is the measurement vector, and *X* is the unknown vector. In a compressed sensing problem, the number of measurements in *P* is much smaller than the number of unknowns in *X*. In other words, the system matrix *A* has more columns than rows. We call the solution *X* sparse if most of the elements in *X* are zero.

Another situation in compressed sensing is that the solution *X* of the system (5) is not sparse; however, a transformed version of *X* is sparse. If such a sparsification transformation operator is denoted by ψ, and the elements in vector *Y*

(6) Y=ψ(X)

are dominated by zeros; then *Y* is sparse. The total count of non-zero elements in *Y* is the $L_{0}$ norm of *Y*. In theory, the solution *X* can be obtained by minimizing the following objective function *F*

(7) $$F={∥AX-P∥}_{L_{2}}^{2}+\alpha {∥\psi (X)∥}_{L_{0^{\prime }}}$$

where α is a tuning parameter.

Let us explain (5) and (7) further. Many real-word problems can be modeled as a system of linear equations (5), where the measurements are represented as a vector *P* and the object being measured is represented by a vector *X*. The measurements *P* usually contain noise. The vector *X* is a discretized version of the real-world object to be estimated. The system matrix *A* describes the first-order approximation of measurement physics. The matrix *A* is assumed to be known. In compressed sensing, the object is under-sampled. Thus, the size of *P* is smaller than the size of *X*, and the matrix *A* has more columns than rows.

The first term in (7), ${∥AX-P∥}_{L_{2}}^{2}$, is the data fidelity term. Minimizing this term is equivalent to solving (5). When the system (5) is under-determined, the solutions in (5) is not unique. The second term in (7), $\alpha {∥\psi (X)∥}_{L_{0}}$, is the Bayesian term. Minimizing this term enforces ψ(X) to be sparse or enforces *X* to be piecewise-constant.

Since the $L_{0}$ norm counts the total number of non-zero elements, it is difficult to use a gradient-based algorithm to minimize the objective function *F* defined in (7). In fact, minimizing the $L_{0}$ norm is an NP-hard problem [11–15], making it computationally infeasible for large-scale problems.

Many researchers attempted to minimize $L_{0}$ norm and found it to be difficult to deal with the $L_{0}$ norm directly [2,3,16–25]. One way is to approximate the $L_{0}$ norm by other norms [2,3,16–19]. Another way is to convert an $L_{0}$-norm minimization problem to an integer programming problem [20,21]. Still another way is to approximate the $L_{0}$-norm by a smooth function [22–25]. The $L_{0}$-norm minimization problem can also be converted to some sub problems by using some thresholds [26,27]. Robitzsch compared an $L_{p}$-norm method with an approximation of the $L_{0}$-norm method and showed that the $L_{0}$-norm method is slightly better [28].

The most popular method to replace the $L_{0}$-norm is the use of the total variation (TV) norm [29–31]. In the TV norm methods, the total variation (TV) norm of the unknown *X* is used to replace the second term in (7). The TV norm minimization method is to use finite difference as the sparsification operator ψ and use the $L_{1}$ norm to approximate the $L_{0}$ norm. If *X* is piecewise constant, the finite difference version is sparse. The $L_{1}$ norm for a vector *X* of *n* elements is given by

(8) $${∥X∥}_{L_{1}}=\sum_{i=1}^{n}\left|x_{i}\right|.$$

Thus, the TV norm of a vector *X* of *n* elements is

(9) $${∥X∥}_{TV}=\sum_{i=1}^{n-1}\left|x_{i}-x_{i-1}\right|.$$

Using the TV norm optimization, the objective function (7) for the vector case becomes

(10) $$F={∥AX-P∥}_{L_{2}}^{2}+\alpha ∥X{∥}_{TV}={∥AX-P∥}_{L_{2}}^{2}+\alpha \sum_{i=1}^{n-1}\left|x_{i}-x_{i-1}\right|.$$

The TV norm of a matrix is not a direct extension of (9) to a higher dimension. We have two different definitions of the TV norm for a two-dimensional matrix (that is, a two-dimensional image) based on the two different ways to define the finite difference [1]. One TV norm is referred to as the isotropic TV norm and the other TV norm is referred to as the anisotropic TV norm. We use double indices for each element of the matrix *X*.

The isotropic TV norm is defined as [1]

(11) $${TV}_{iso}(X)=\sum_{i,j}\sqrt{{\left(x_{i+1,j}-x_{i,j}\right)}^{2}+{\left(x_{i,j+1}-x_{i,j}\right)}^{2}},$$

and the anisotropic TV norm is defined by [1]

(12) $${TV}_{\mathrm{aniso}}(X)=\sum_{i,j}\left(\left|x_{i+1,j}-x_{i,j}\right|+\left|x_{i,j+1}-x_{i,j}\right|\right).$$

For the two-dimensional image case, the objective function (10) can then be written as

(13) $$F={∥AX-P∥}_{L_{2}}^{2}+\alpha {TV}_{iso}(X)={∥AX-P∥}_{L_{2}}^{2}+\alpha \sum_{i,j}\sqrt{{\left(x_{i+1,j}-x_{i,j}\right)}^{2}+{\left(x_{i,j+1}-x_{i,j}\right)}^{2}}$$

and

(14) $$F={∥AX-P∥}_{L_{2}}^{2}+\alpha {TV}_{\mathrm{aniso}}(X)={∥AX-P∥}_{L_{2}}^{2}+\alpha \sum_{i,j}\left(\left|x_{i+1,j}-x_{i,j}\right|+\left|x_{i,j+1}-x_{i,j}\right|\right),$$

respectively.

**Figure 1.**
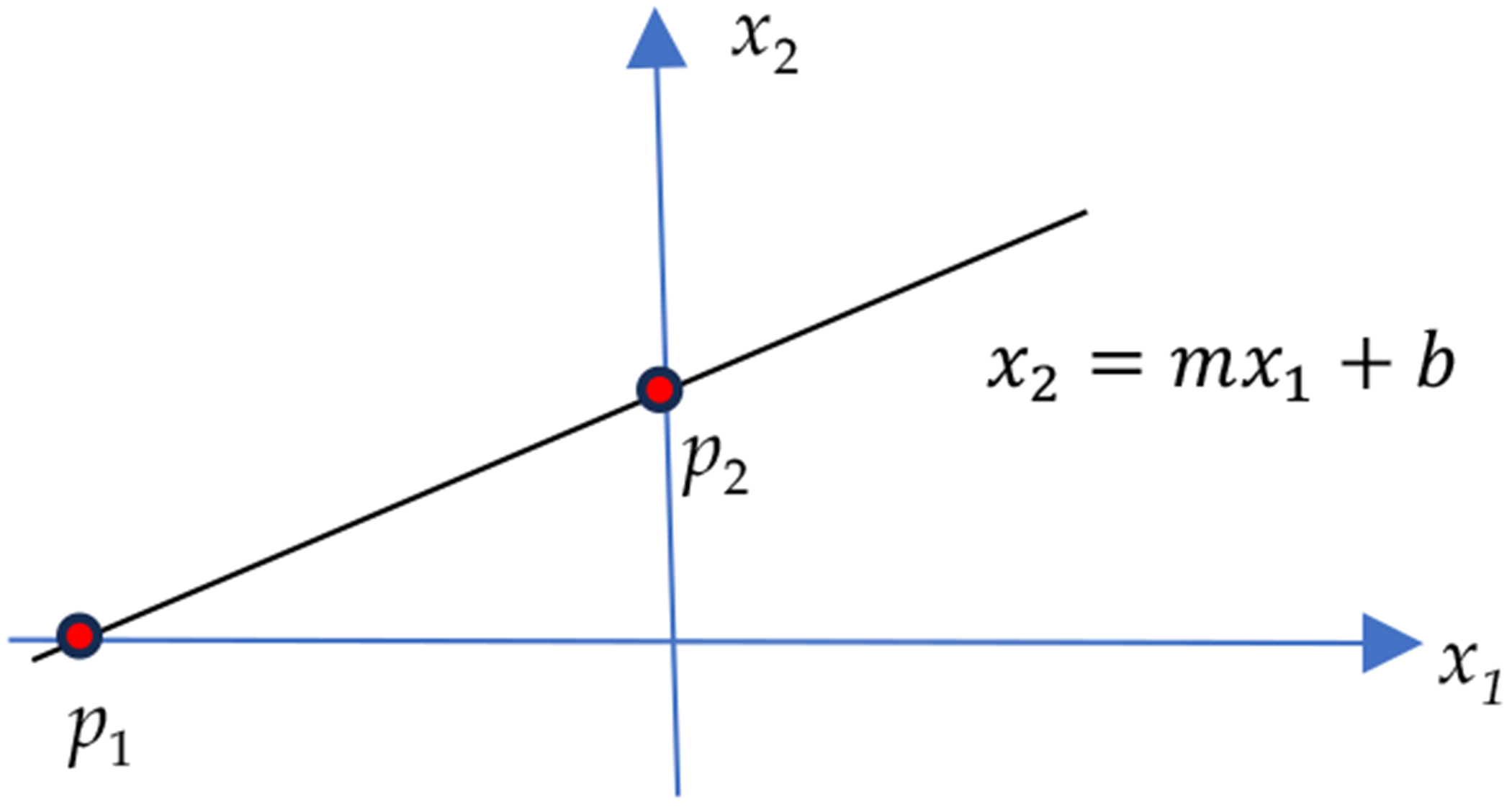
Points ($x_{1},x_{2}$) on the line $x_{2}={\mathrm{mx}}_{1}+b$ are solutions to (1).

The justification of using $L_{1}$ norm to approximate the $L_{0}$ norm is that they both prefer a solution with more zeros. Figure 1 shows an exemplary solution line $x_{2}=mx_{1}+b$; any point ($x_{1},x_{2}$) on this line is a solution to (5). This solution line may intersect the coordinate axes at two points: $p_{1}$ and $p_{2}$, with $p_{1}=(-b/m,0)$ and $p_{2}=(0,b)$, respectively. The $L_{0}$ minimization method will select either $p_{1}$ or $p_{2}$ to be the solution, because ${∥p_{1}∥}_{L_{0}}={∥p_{2}∥}_{L_{0}}=1$ and other points on the line have a larger $L_{0}$ norm of ${∥\left(x_{1},x_{2}\right)∥}_{L_{0}}=2$. As for the $L_{1}$ norm, ${∥p_{1}∥}_{L_{1}}=|b/m|$, and ${∥p_{2}∥}_{L_{1}}=|b|$. The $L_{1}$ minimization method will select $p_{1}$ if |m|>1 or $p_{2}$ if |m|<1 to be the solution. The $L_{1}$ minimization method will select either $p_{1}$ or $p_{2}$ to be the solution if |m|=1. Therefore, $L_{0}$ minimization and $L_{1}$ minimization may select the same solutions. The justification illustrated in our toy example is unrealistic because we do not have the luxury to obtain this solution line to search along.

In the objective function (10), the first term involving the $L_{2}$ norm is dominating. Thus, when the parameter α is small, the optimal solution of (10) may not make ψ(X) sparse.

**Figure 2.**
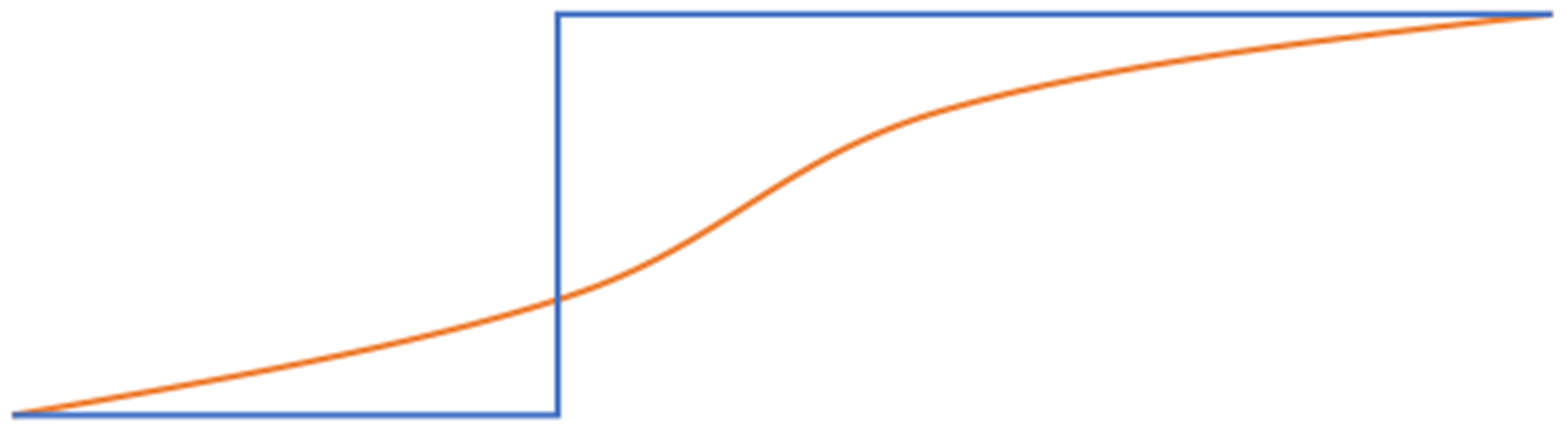
Two curves with the same TV value but different finite-difference plus $L_{0}$ values.

A drawback of the TV norm is that it cannot tell the difference between a smooth monotonic transition and a sharp monotonic transition (See Figure 2).

Therefore, using the $L_{0}$ norm and objective functions (7) may work better than objective functions (13) and (14), in the sense that the $L_{0}$ norm can tell the difference for the two curves shown in Figure 2. A drawback of the finite difference using the $L_{0}$ method is that we do not have an effective and efficient way to minimize the $L_{0}$ norm. The main goal of the paper is to directly minimize the objective function (7) with the use of the $L_{0}$ norm and find an innovative way to handle the $L_{0}$ norm.

## 2. Methods

In Section 1, we analyzed the drawbacks of the TV norm and the $L_{0}$ norms. In this section, we will develop a method to replace the TV norm by the $L_{0}$ norm for a practical iterative algorithm.

**Figure 3.**
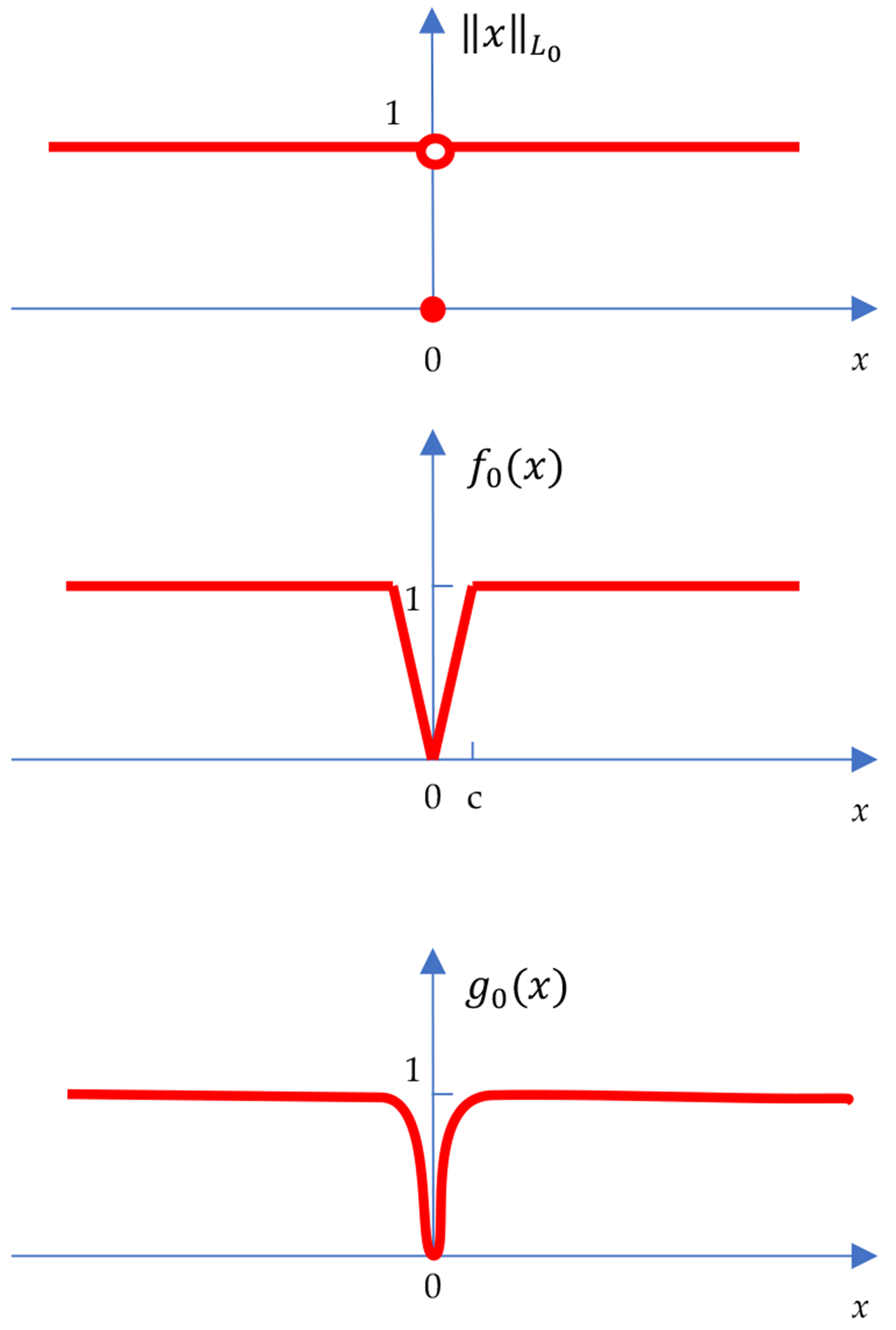
Three ways to re-define the $L_{0}$ norm for a scalar *x*. **Top**: The traditional definition (1). **Middle**: The proposed definition (15). **Bottom**: A smooth version of (15).

We notice from the traditional definition of the $L_{0}$ norm (see Figure 3 Top)

$${∥x∥}_{L_{0}}=\left\{\begin{array}{lll} 0 & \mathrm{if} & x=0\\ 1 & \mathrm{if} & x\neq 0. \end{array}\right.$$

that the “if *x* = 0” statement is hardly true in a practical computer algorithm, because in practice a very small value (for example, an image pixel value of 0.000000000001) can be treated as zero. For the scalar case, it is reasonable to replace the definition (1) by (15) below

(15) $$f_{0}(x)=\left\{\begin{array}{lll} \frac{\left|x\right|}{c} & \mathrm{if} & |x|\leq c\\ 1 & \mathrm{if} & |x|>c. \end{array}\right.$$

for a chosen c>0. In (15), c is a tuning parameter, determined by trial-and-error. This modification is illustrated in Figure 3 (Middle). The piecewise linear function $f_{0}(x)$ defined in (5) is continuous and is a good approximation of ${∥x∥}_{L_{0}}$ when c→0.

In fact, we can have many versions of the $L_{0}$ norm. For example, the version shown in Figure 3 (Bottom) is a smooth function; the derivative of the curve exists everywhere. One such smooth function is

$$g_{0}(x)=1-e^{-x^{2}/c}$$

for a small c>0.

The requirement to be differentiable everywhere is not needed. We believe that a gradient-based optimization algorithm only requires the existence of the left and right derivatives everywhere. Let us consider a toy example of

(16) f(x)=|x|

We want to find the minimum of the function defined in (16). It is obvious that the solution is

(17) x=0

We notice that the function f(x)=|x| is not differentiable at x=0. At x=0, the left derivative and right derivative of f(x) are $f_{\mathrm{left}}^{\prime }(0)=-1$ and $f_{\mathrm{right}}^{\prime }(0)=1$, respectively. A gradient-based optimization algorithm can be crafted as

(18) $$x^{\mathrm{next}}=x^{\mathrm{current}}-\lambda \frac{f_{\mathrm{left}}^{\prime }\left(x^{\mathrm{current}}\right)+f_{\mathrm{right}}^{\prime }\left(x^{\mathrm{current}}\right)}{2}$$

where the parameter λ>0 controls the step size of the iterative optimization algorithm. Algorithm (18) is nothing but the commonly used gradient descent algorithm, except that the gradient is replaced by the average of the left and right gradients. Therefore, our definition of the $L_{0}$ norm is user-friendly in gradient-based iterative optimization algorithms. Using a smooth function $g_{0}(x)$ shown in Figure 3 (Bottom), to approximate the $L_{0}$ norm is not necessary.

To extend the revised $L_{0}$ definition from the scalar case (15) to a matrix *X*, whose elements are denoted as $x_{i,j}$, we have

(19) $$f_{0}(X)=\sum_{i,j}f_{0}\left(x_{i,j}\right)$$

where $f_{0}\left(x_{i,j}\right)$ is defined in (15). The definition of (19) is still not effective in a gradient-based iterative optimization algorithm. The partial derivative of $f_{0}(X)$ with respect to $x_{i,j}$ is calculated as

(20) $$\frac{\partial f_{0}(X)}{\partial x_{i,j}}=\frac{df_{0}(X)}{dx_{i,j}}=\left\{\begin{array}{ccc} \frac{1}{c}sgn\left(x_{i,j}\right) & \mathrm{if} & \left|x_{i,j}\right|\leq c\\ 0 & \mathrm{if} & \left|x_{i,j}\right|>c. \end{array}\right.$$

Let

(21) $$step(x)=\left\{\begin{array}{lll} 1 & \mathrm{if} & x\geq 0\\ 0 & \mathrm{if} & x<0. \end{array}\right.$$

Then (20) can be expressed as

(22) $$\frac{\partial f_{0}(X)}{\partial x_{i,j}}=\frac{df_{0}(X)}{dx_{i,j}}=\frac{1}{c}\times sgn\left(x_{i,j}\right)\times step\left(c-\left|x_{i,j}\right|\right).$$

**Figure 4.**
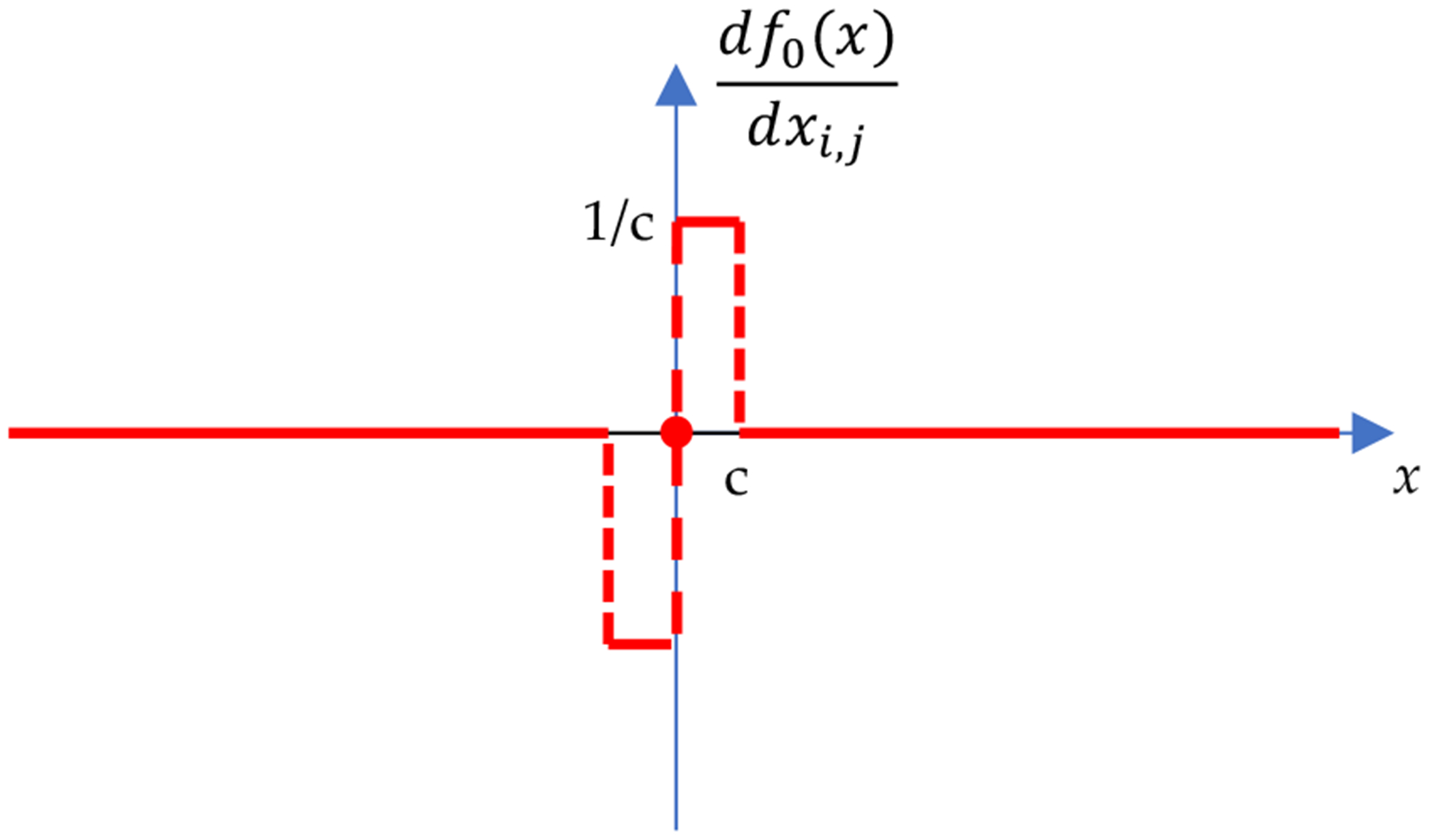
The curve of $f_{0}(x)/dx$ according to the new definition (15).

The plot of (22) is shown in Figure 4. It is observed that when $\left|x\right|>c$ the derivative is 0. In other words, there will be no update action in the iterative algorithm for most of the image pixels. This makes the optimization algorithm almost inactive and ineffective.

**Figure 5.**
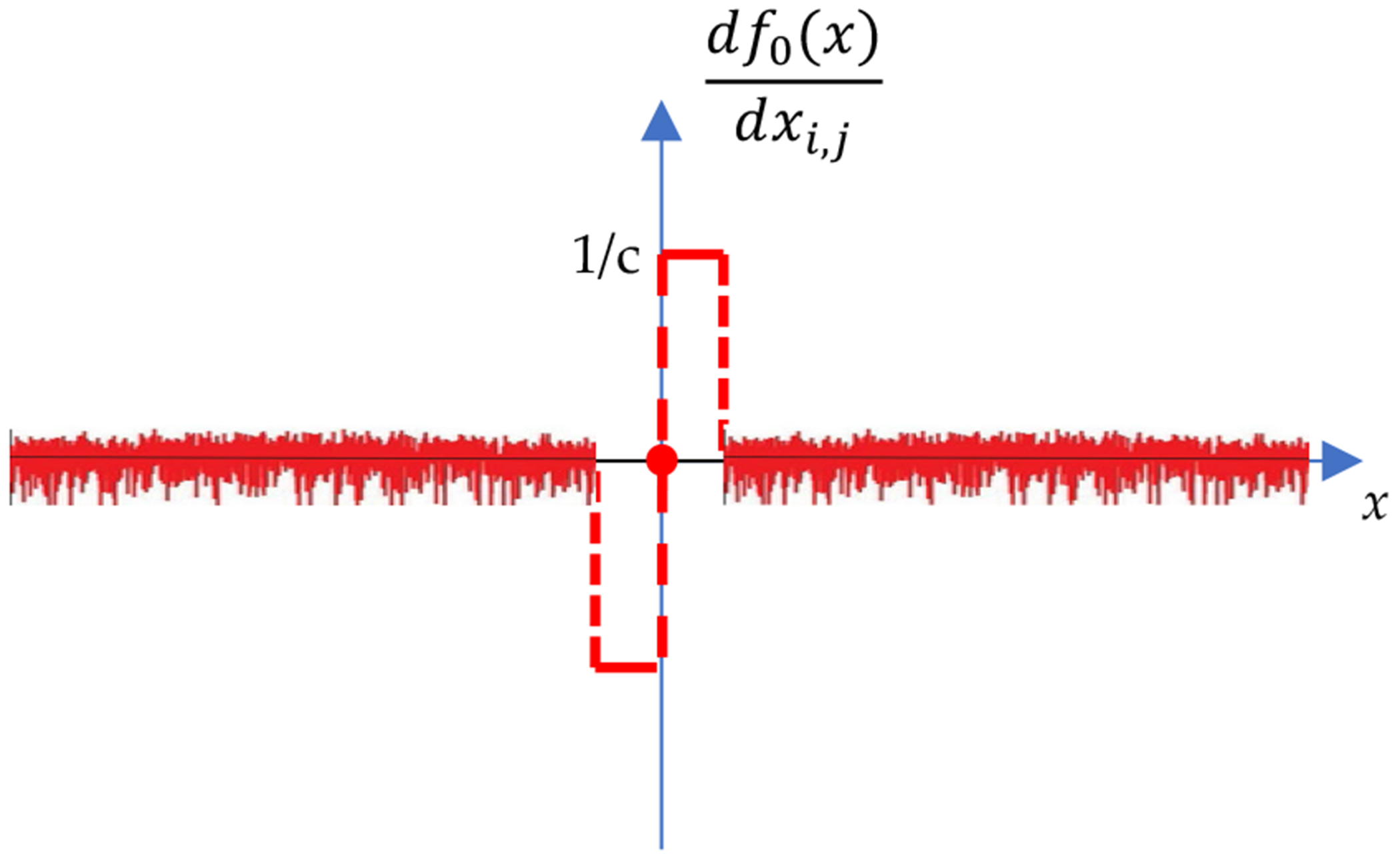
The curve of $f_{0}(x)/dx$ according to the new definition (15) and by replacing the zeros with zero-mean random variables.

In order to obtain more actions in the iterative algorithm, our next innovation is to replace the zero values in $df_{0}(x)/dx$ by small zero-mean random variables. Thus, Figure 4 becomes Figure 5. Since the optimization algorithms need the expression of $df_{0}(x)/dx$ and do not care if there is a corresponding expression of $f_{0}(x)$, we do not bother to investigate what the definition of $f_{0}(x)$ is corresponding to the $df_{0}(x)/dx$ shown in Figure 5.

The mathematical expression for the revised derivative shown in Figure 5 is given as

(23) $$\frac{\partial f_{0}(X)}{\partial x_{i,j}}=\frac{df_{0}(X)}{dx_{i,j}}=\frac{1}{c}\times sgn\left(x_{i,j}\right)\times step\left(c-\left|x_{i,j}\right|\right)+rand\times step\left(\left|x_{i,j}\right|-c\right),$$

where *rand* is a small zero-mean random variable. The tuning parameter *c* is selected by trail-and-error, depending on the application.

We remind the reader that our images are not sparse, but piecewise constant. We need a sparsification transformation operator ψ to convert a piecewise-constant image to a sparse image. We chose the finite difference operator as the sparsification transformation operator ψ. Just like cases of isotropic TV (11) and anisotropic (12) TV definitions, we can have two definitions of $\partial {∥x∥}_{L_{0}}/\partial x_{i,j}$, one being the isotropic derivative version and the other being the anisotropic version.

The isotropic version is defined as

(24) $$\;\frac{\partial {∥x∥}_{L_{0}}}{\partial x_{i,j}}=\frac{\partial \sum_{n,m}{∥\sqrt{{\left(x_{n+1,m}-x_{n,m}\right)}^{2}+{\left(x_{n,m+1}-x_{n,m}\right)}^{2}}∥}_{L_{0}}}{\partial x_{i,j}}\;=\sum_{n,m}\frac{\partial {∥\sqrt{{\left(x_{n+1,m}-x_{n,m}\right)}^{2}+{\left(x_{n,m+1}-x_{n,m}\right)}^{2}}∥}_{L_{0}}}{\partial \sqrt{{\left(x_{n+1,m}-x_{n,m}\right)}^{2}+{\left(x_{n,m+1}-x_{n,m}\right)}^{2}}}\frac{\partial \sqrt{{\left(x_{n+1,m}-x_{n,m}\right)}^{2}+{\left(x_{n,m+1}-x_{n,m}\right)}^{2}}}{\partial x_{i,j}}\;\;=\sum_{n,m}g(n,m)\times \frac{\partial \sqrt{{\left(x_{n+1,m}-x_{n,m}\right)}^{2}+{\left(x_{n,m+1}-x_{n,m}\right)}^{2}}}{\partial x_{i,j}}\;\;=g(i-1,j)\frac{\partial \sqrt{{\left(x_{i,j}-x_{i-1,j}\right)}^{2}+{\left(x_{i-1,j+1}-x_{i-1,j}\right)}^{2}}}{\partial x_{i,j}}\;\;+g(i,j)\frac{\partial \sqrt{{\left(x_{i+1,j}-x_{i,j}\right)}^{2}+{\left(x_{i,j+1}-x_{i,j}\right)}^{2}}}{\partial x_{i,j}}\;\;+g(i,j-1)\frac{\partial \sqrt{{\left(x_{i+1,j-1}-x_{i,j-1}\right)}^{2}+{\left(x_{i,j}-x_{i,j-1}\right)}^{2}}}{\partial x_{i,j}}\;\;=g(i-1,j)\frac{x_{i,j}-x_{i-1,j}}{\sqrt{{\left(x_{i,j}-x_{i-1,j}\right)}^{2}+{\left(x_{i-1,j+1}-x_{i-1,j}\right)}^{2}}}\;\;+g(i,j-1)\frac{x_{i,j}-x_{i,j-1}}{\sqrt{{\left(x_{i+1,j-1}-x_{i,j-1}\right)}^{2}+{\left(x_{i,j}-x_{i,j-1}\right)}^{2}}}\;\;-g(i,j)\frac{x_{i+1,j}+x_{i,j+1}-2x_{i,j}}{\sqrt{{\left(x_{i+1,j}-x_{i,j}\right)}^{2}+{\left(x_{i,j+1}-x_{i,j}\right)}^{2}}}\;\;=g(i-1,j)\frac{x_{i,j}-x_{i-1,j}}{u(i-1,j)}+g(i,j-1)\frac{x_{i,j}-x_{i,j-1}}{u(i,j-1)}-g(i,j)\frac{x_{i+1,j}+x_{i,j+1}-2x_{i,j}}{u(i,j)}$$

with

(25) $$u(n,m)=\sqrt{{\left(x_{n+1,m}-x_{n,m}\right)}^{2}+{\left(x_{n,m+1}-x_{n,m}\right)}^{2}}$$

and

(26) $$\;g(n,m)=\frac{\partial {∥\sqrt{{\left(x_{n+1,m}-x_{n,m}\right)}^{2}+{\left(x_{n,m+1}-x_{n,m}\right)}^{2}}∥}_{L_{0}}}{\partial \sqrt{{\left(x_{n+1,m}-x_{n,m}\right)}^{2}+{\left(x_{n,m+1}-x_{n,m}\right)}^{2}}}\;\;=\frac{1}{c}\times step\left(c-\sqrt{{\left(x_{n+1,m}-x_{n,m}\right)}^{2}+{\left(x_{n,m+1}-x_{n,m}\right)}^{2}}\right)\;+rand\times step\left(\sqrt{{\left(x_{n+1,m}-x_{n,m}\right)}^{2}+{\left(x_{n,m+1}-x_{n,m}\right)}^{2}}-c\right)\;\;=\frac{1}{c}\times step(c-u(n,m))+rand\times step(u(n,m)-c).$$

The anisotropic version is defined by

(27) $$\;\frac{\partial {∥x∥}_{L_{0}}}{\partial x_{i,j}}=\frac{\partial \sum_{n,m}\left({∥x_{n+1,m}-x_{n,m}∥}_{L_{0}}+{∥x_{n,m+1}-x_{n,m}∥}_{L_{0}}\right)}{\partial x_{i,j}}\;\;=\frac{\partial \left({∥x_{i,j}-x_{i-1,j}∥}_{L_{0}}+{∥x_{i-1,j+1}-x_{i-1,j}∥}_{L_{0}}\right)}{\partial x_{i,j}}\;\;+\frac{\partial \left({∥x_{i+1,j}-x_{i,j}∥}_{L_{0}}+{∥x_{i,j+1}-x_{i,j}∥}_{L_{0}}\right)}{\partial x_{i,j}}\;\;+\frac{\partial \left({∥x_{i+1,j-1}-x_{i,j-1}∥}_{L_{0}}+{∥x_{i,j}-x_{i,j-1}∥}_{L_{0}}\right)}{\partial x_{i,j}}\;=\frac{\partial \left({∥x_{i,j}-x_{i-1,j}∥}_{L_{0}}\right)}{\partial x_{i,j}}+\frac{\partial \left({∥x_{i+1,j}-x_{i,j}∥}_{L_{0}}\right)}{\partial x_{i,j}}+\frac{\partial \left({∥x_{i,j+1}-x_{i,j}∥}_{L_{0}}\right)}{\partial x_{i,j}}+\frac{\partial \left({∥x_{i,j}-x_{i,j-1}∥}_{L_{0}}\right)}{\partial x_{i,j}}\;\;=\frac{1}{c}\times sgn\left(x_{i,j}-x_{i-1,j}\right)\times step\left(c-\left|x_{i,j}-x_{i-1,j}\right|\right)+{rand}_{1}\times step\left(\left|x_{i,j}-x_{i-1,j}\right|-c\right)\;\;+\frac{1}{c}\times sgn\left(x_{i+1,j}-x_{i,j}\right)\times step\left(c-\left|x_{i+1,j}-x_{i,j}\right|\right)+{rand}_{2}\times step\left(\left|x_{i+1,j}-x_{i,j}\right|-c\right)\;\;+\frac{1}{c}\times sgn\left(x_{i,j+1}-x_{i,j}\right)\times step\left(c-\left|x_{i,j+1}-x_{i,j}\right|\right)+{rand}_{3}\times step\left(\left|x_{i,j+1}-x_{i,j}\right|-c\right)\;\;+\frac{1}{c}\times sgn\left(x_{i,j}-x_{i,j-1}\right)\times step\left(c-\left|x_{i,j}-x_{i,j-1}\right|\right)+{rand}_{4}\times step\left(\left|x_{i,j}-x_{i,j-1}\right|-c\right)$$

If a gradient based iterative algorithm is used to minimize an objective function, the algorithm does not update the image pixel $x_{i,j}$ when the partial derivative of the objective function with respect to $x_{i,j}$ is zero. More often than not, the L_0_ Bayesian term is ‘silent’ and has little contribution to find a piecewise constant solution. If we replace zero with zero-mean random variables, we actually disturb the algorithm so that it is not ‘silent.’

This strategy is similar to the simulated annealing algorithm, which generates random solutions and gradually rejects less optimal solutions [32]. However, there is no theoretical guarantee that random solutions are better solutions.

By replacing zero by zero-mean random variables does not change the fact that the L_0_ defined norm is not convex. To obtain the global minimum, one must do exhaustive search for the entire solution space. In other words, L_0_ minimization even with the current modification is still NP-hard. The proposed algorithm is gradient based, does not do exhaustive search, and usually does not reach the global minimum.

A drawback of the objective function (7) is the difficulty in selecting the control parameter α. Instead of minimizing the objective function (7) directly, we propose to alternatively minimize the data fidelity term ${∥AX-P∥}_{L_{2}}^{2}$ and the Bayesian term ${∥\psi (X)∥}_{L_{0}}$. In this way, the value of tuning parameter α is no longer important. In other words, we use an iterative Projection onto Convex Sets (POCS) algorithm.

There are many algorithms to minimize the first term (i.e., the data fidelity term). We chose the maximum likelihood expectation maximization (MLEM) algorithm in our implementation [33–37]. On the other hand, the Bayesian term is minimized by a gradient descent algorithm. For an image reconstruction task, at the pixel $x_{i,j}$, the gradient is given by (27).

**Figure 6.**
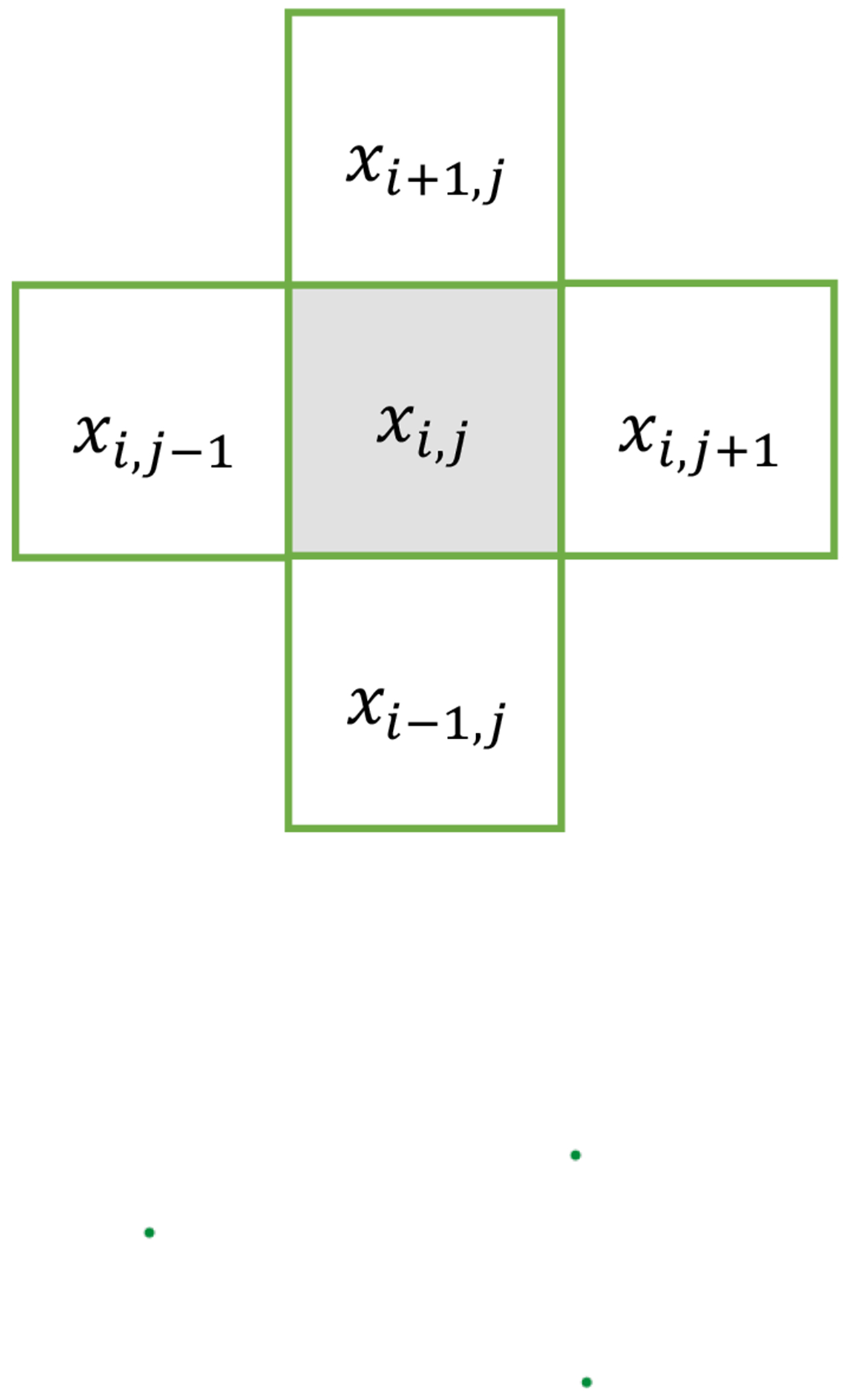
We consider updating a pixel $x_{i,j}$ by calculating the gradients with its horizonal and vertical neighbors.

Now, we use a different way to explain the algorithm to enforce the $L_{0}$ norm of the gradient image. For an image reconstruction task, we want to update the pixel $x_{i,j}$, as shown in Figure 6. The horizontal and vertical gradients of pixel $x_{i,j}$ are $x_{i,j}-x_{i-1,j},x_{i,j}-x_{i+1,j}$, and $x_{i,j}-x_{i,j-1},x_{i,j}-x_{i,j+1}$, correspondingly. We want to minimize the $L_{0}$ norms of these four differences. The derivative of these four $L_{0}$ norms have this common expression:

(28) $$D(x_{i,j},x_{\mathrm{neighbor}})=\mathrm{Derivative}=a\times sgn(x_{i,j}-x_{\mathrm{neighbor}})$$

where

(29) $$a=\left\{\begin{array}{cc} 1/c, & \mathrm{when}\\ \mathrm{rand,} & \mathrm{when} \end{array}\left|\begin{array}{l} x_{i,j}-x_{\mathrm{neighbor}}|<c,\\ x_{i,j}-x_{\mathrm{neighbor}}|\geq c. \end{array}\right.\right.$$

A gradient descent algorithm to update $x_{i,j}$ is given as

(30) $$x_{i,j}^{next}=x_{i,j}-\mu \left[D\left(x_{i,j},x_{i,j-1}\right)+D\left(x_{i,j},x_{i,j+1}\right)+D\left(x_{i,j},x_{i,j-1}\right)+D\left(x_{i,j},x_{i,j+1}\right)\right]$$

Here, the zero-mean random noise, rand, is uniformly distributed in [−1, 1]. The tuning parameter *c* was chosen as 0.1 by trial-and-error in our implementation.

In our computer simulations, we used 100,000 iterations of the POCS algorithm. At each POCS iteration, we first ran 10 iterations of the MLEM algorithm to minimize the data fidelity term and then we ran 10 iterations of the gradient descent algorithm to minimize the $L_{0}$ term with a step size of 0.1.

**Figure 7.**
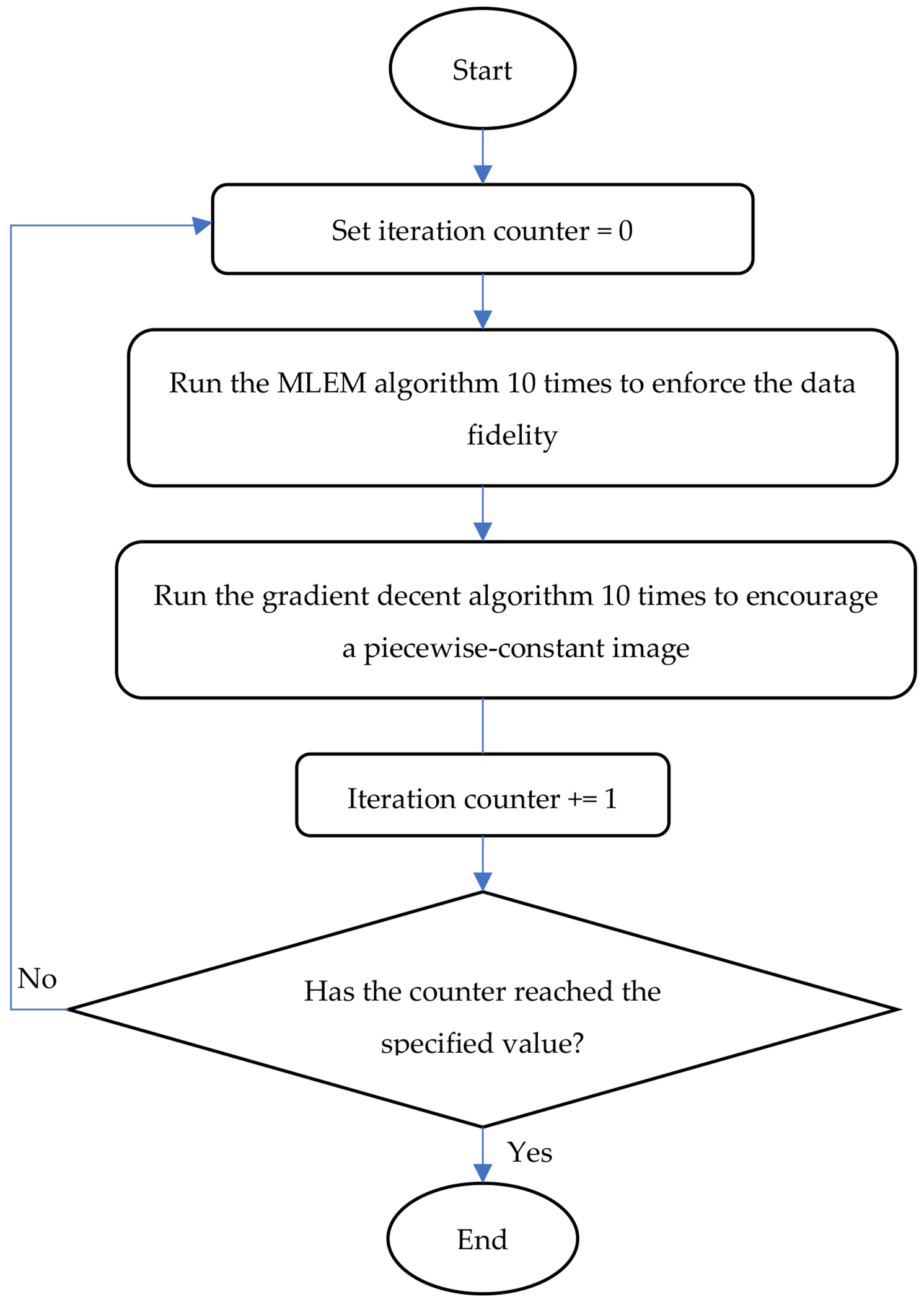
The flowchart of the proposed POCS algorithm.

We summarize the main steps in the development of the proposed algorithm as follows. We started with the original $L_{0}$ definition (1). Then, this expression was replaced by a piecewise-linear continuous approximation (15). Next, the partial derivative (22) was replaced by (23). The proposed POCS algorithm is summarized as a flowchart in Figure 7.

## 3. Results

We applied the proposed POCS algorithm to a limited-angle two-dimensional image reconstruction problem. The computer-generated phantoms were considered. The first phantom had a large, uniform disk as the background and 13 smaller, uniform squares and disks. The sizes, locations, and intensities for each square and disk are listed in Table 1. The second phantom was the famous Shepp–Logan head phantom [38]. No noise was added to the phantom projection data.

**Figure 8.**
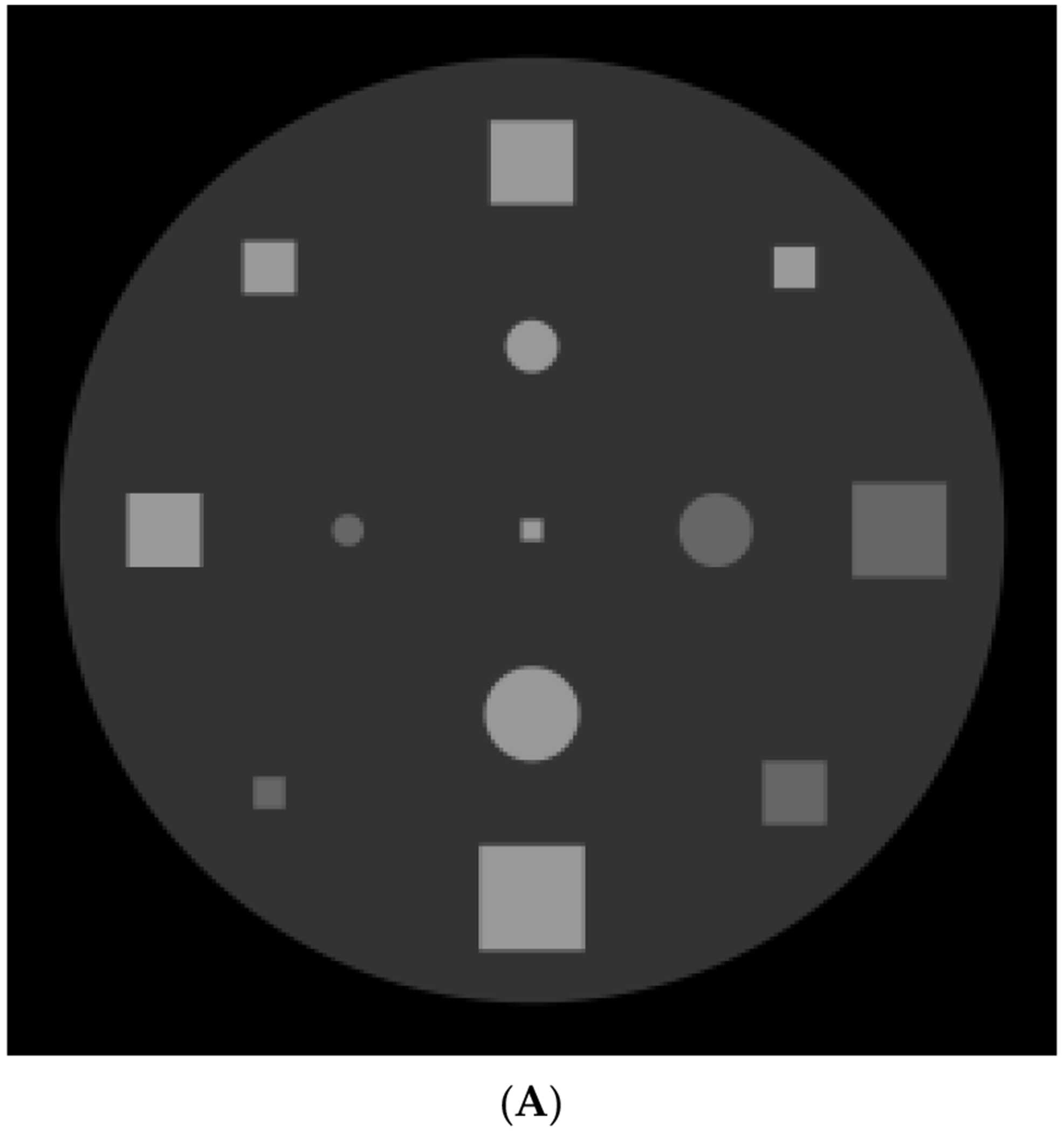

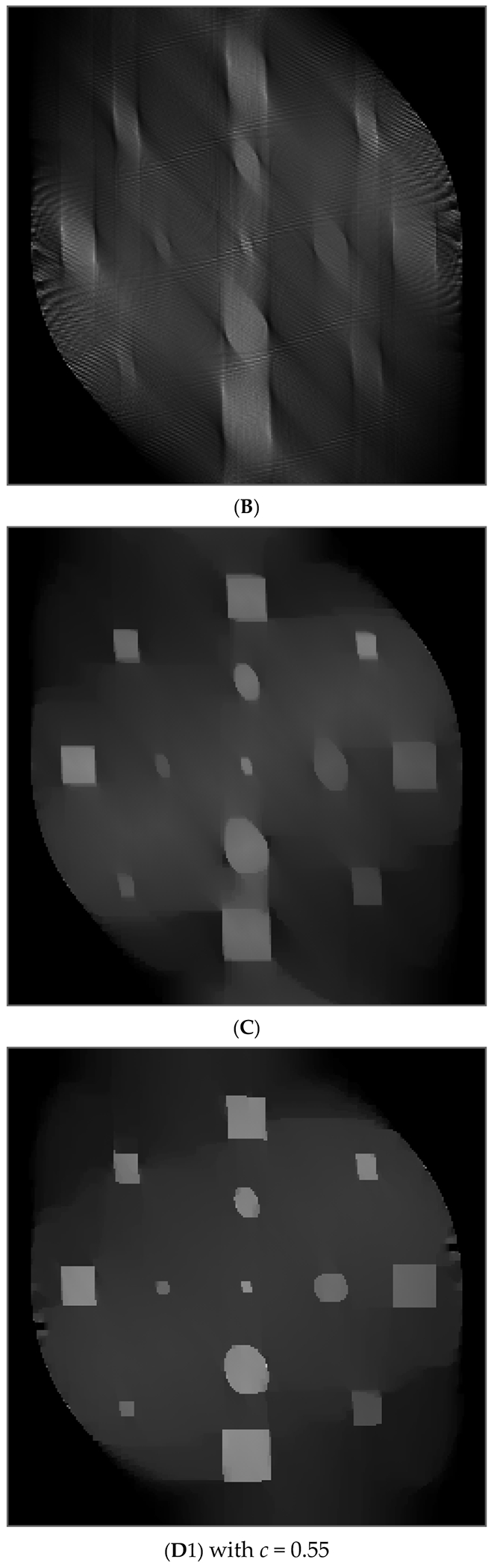

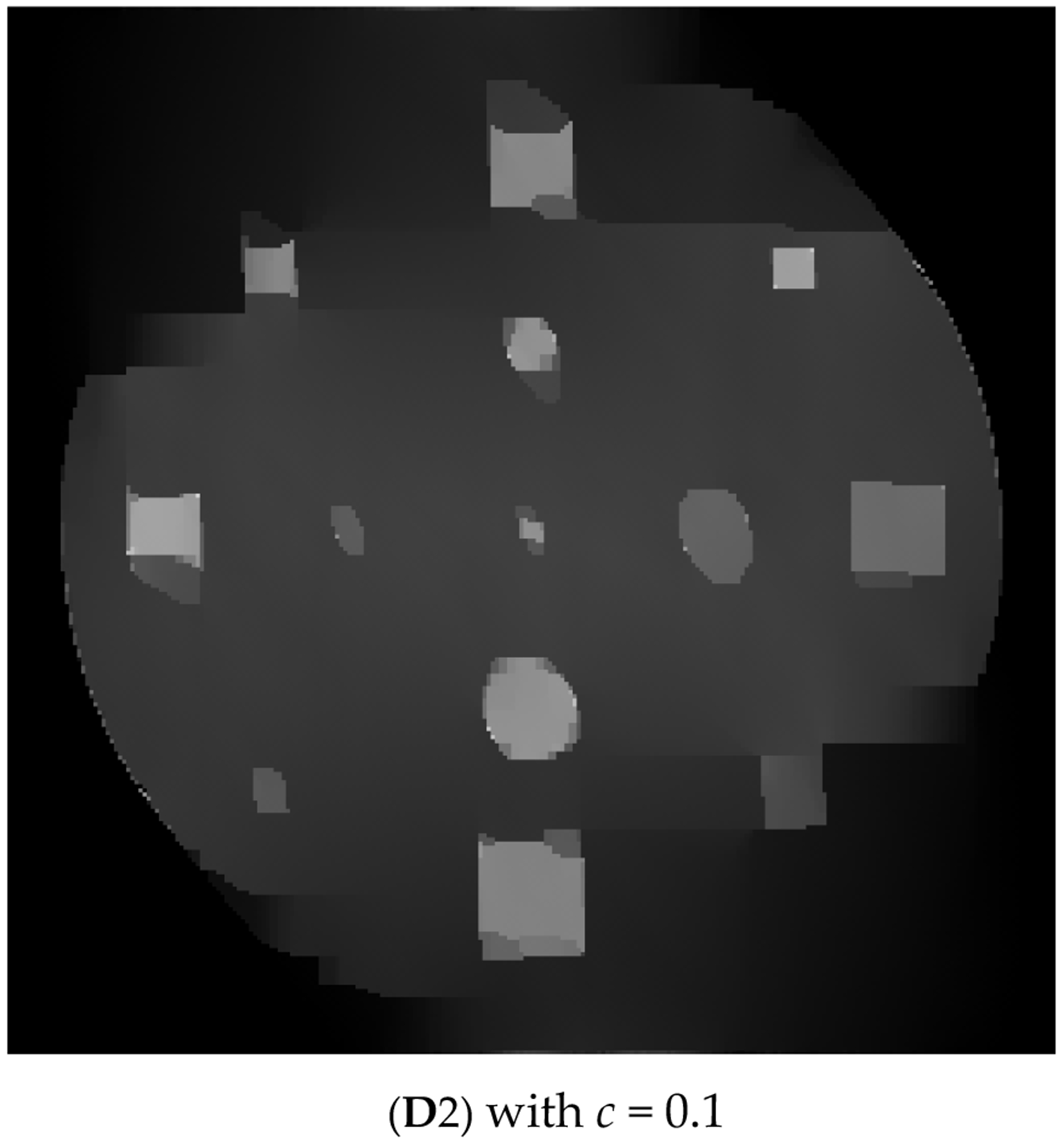
Results of the first phantom study. (**A**) True phantom; (**B**) MLEM reconstruction; (**C**) TV reconstruction; (**D**1, **D**2) Proposed revised $L_{0}$ norm reconstruction.

**Figure 9.**
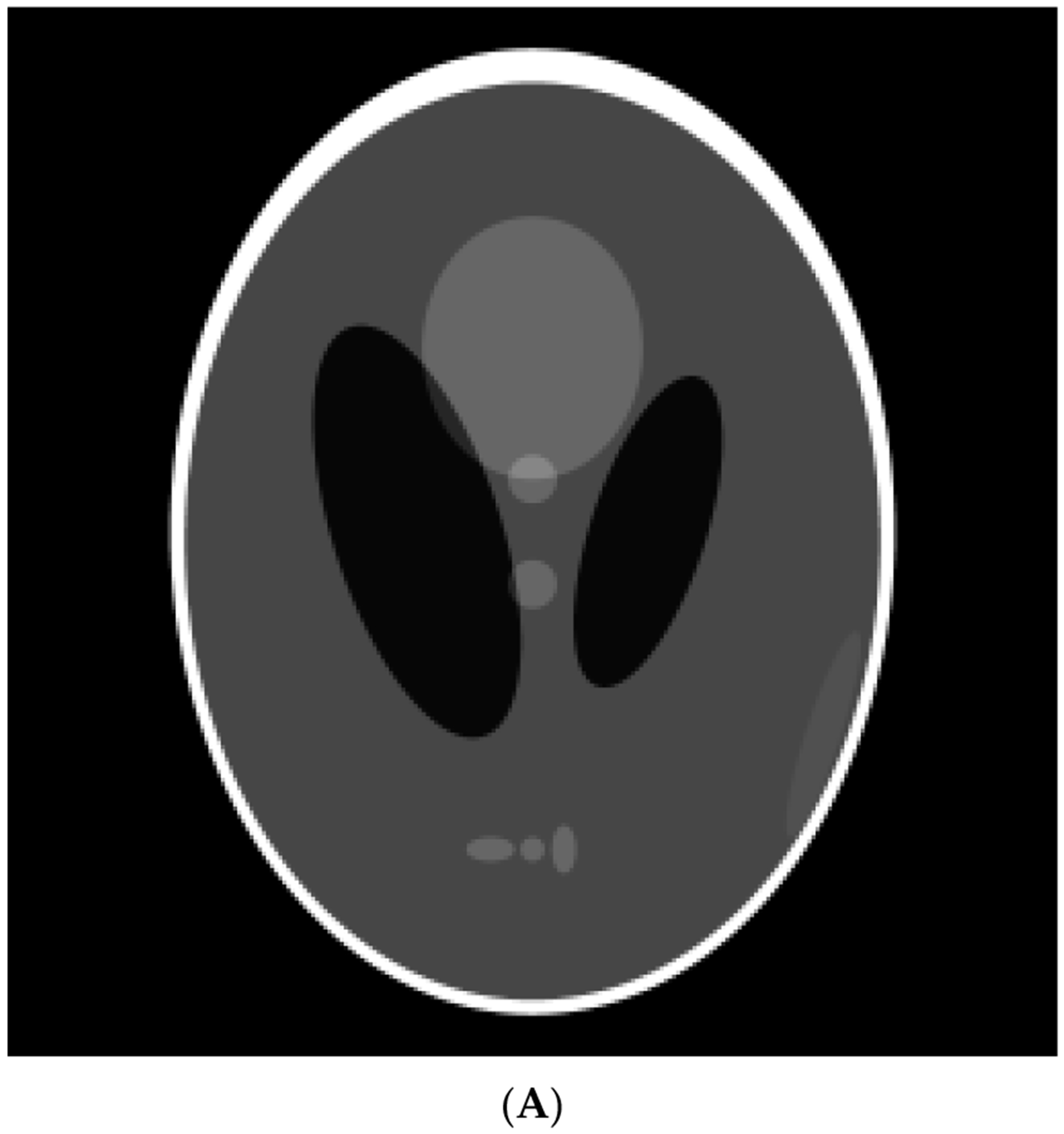

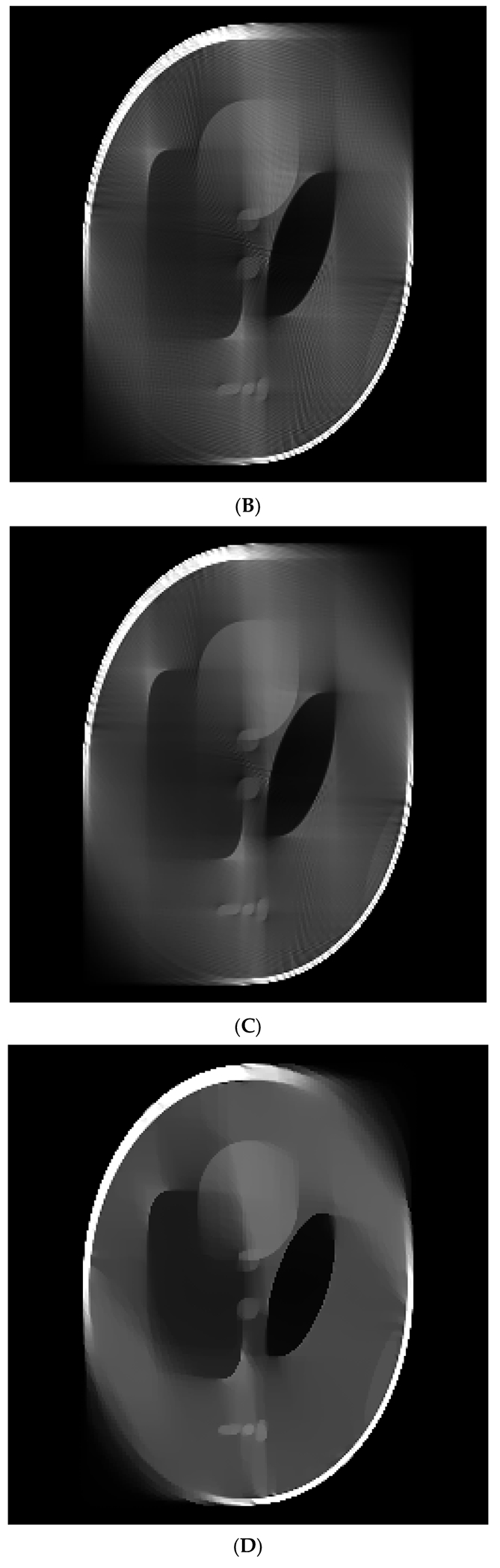
Results of the second phantom study. (**A**) True phantom; (**B**) MLEM reconstruction; (**C**) TV reconstruction; (**D**) Proposed revised $L_{0}$ norm reconstruction.

In the computer simulations, the parallel-beam imaging geometry was considered. The scanning angle for the first phantom was 40°, and for the second phantom was 90°. The image size was 256 × 256. For these two phantom studies, three image reconstruction algorithms were compared in Figures 8 and 9, respectively: The well-known MLEM algorithm [34], the MLEM-TV algorithm [33], and the proposed POCS revised $L_{0}$-norm minimization algorithm.

The iteration number was 400 in the POCS algorithm. Within each iteration, there were 10 iterations of the MLEM algorithm for data fidelity enforcement and 10 iterations of the gradient decent algorithm for piecewise-constant enforcement.

The only tuning parameter in the MLEM algorithm is the number of iterations. However, the gradient descent algorithm has two tuning parameters: the number of iterations and the step size. The step size was chosen as 0.0001 in the first phantom reconstruction and was 0.00001 in the second phantom reconstruction.

The MLEM reconstruction has the most limited-angle artifacts, and the shapes of the small objects are not well defined. The TV reconstruction slightly improves the boundaries of the small objects in the image. The most significant improvement is achieved by the proposed POCS $L_{0}$ minimization algorithm.

Tables 2 and 3 show the quantitative evaluation results with the structural similarity (SSIM) [39] and the peak signal-to-noise ratio (PSNR) [40] for the two phantom studies, respectively. An SSIM value closer to 1 indicates better image quality. A greater PSNR value indicates better image quality.

We observe that the reconstruction artifacts depend heavily on the size and the orientation of an object. A larger object tends to have more severe artifacts. If there are many smaller objects inside a larger object, the artifacts from each smaller object interact. Therefore, the distance between objects affects the overall artifacts. In the first phantom, the small objects are isolated from each other. In the second phantom, the small objects are close to each other. The second phantom is more difficult to reconstruct than the first phantom.

## 4. Conclusions

The TV norm has a drawback that it cannot distinguish a smooth function and a piecewise-constant function. The TV Bayesian objective function may not be effective in promoting a piecewise constant solution. The $L_{0}$ norm, on the other hand, is difficult to implement in a gradient-based optimization algorithm. This paper aims to address these drawbacks.

The difficulty of using the L_0_ norm in an optimization algorithm is well known. Remedies have been proposed the tested by many researchers. The efforts can be classified into two categories: approximating the L_0_ norm by a different norm and approximating the L_0_ norm itself by a continuous functional. The well-known TV-norm optimization belongs to the first category. Our paper belongs to the second category. A unique feature of our method is in the region where the signal is not zero. In this region, the traditional L_0_ norm has $\frac{\partial {∥x∥}_{L_{0}}}{\partial x_{i,j}}=0$. We replace this 0 with a zero-mean random variable. There are many methods that combine L_0_, L_1_, and TV models. The unique feature of our method is the introduction of randomness in the derivative.

The L_0_ norm is not convex. The gradient-based algorithm usually does not converge to the global minimum. We introduce a zero-mean random perturbation to the algorithm; this random perturbation gives the algorithm a chance to ‘jump out’ from a local minimum to another local minimum with a smaller objective function value.

In this paper, we replace the Bayesian algorithm with a POCS algorithm and revise the derivative of the $L_{0}$ norm so that it does not have a constant zero in most cases. As an application to limited-angle tomography, the proposed algorithm outperforms the MLEM-TV algorithm when the scanning angular range is small.

It is difficult to compare any two algorithms in general, because the performance of the algorithms is application-dependent. As shown in our two phantom studies, different applications may require different minimal scanning angular ranges.

## Figures and Tables

**Table 1.** Parameters for the first phantom.

Type	Center x	Center y	Diameter or Side Length	Rotation Angle	Density
Circle	0	0	230.40	0	0.5
Square	89.60	0	25.60	0	1.0
Square	0	89.60	23.04	0	0.5
Square	−89.60	0	20.48	0	1.0
Square	0	−89.60	17.92	0	1.0
Square	64.00	64.00	15.36	0	0.5
Square	−64.00	−64.00	12.80	0	1.0
Square	−64.00	64.00	10.24	0	1.0
Square	64.00	−64.00	7.68	0	0.5
Square	0	0	5.12	0	1.0
Circle	44.80	0	23.04	0	1.0
Circle	0	44.80	17.92	0	0.5
Circle	−44.80	0	12.80	0	1.0
Circle	0	−44.80	7.68	0	0.5

**Table 2.** Quantitative evaluation results for the first phantom.

Method	PSNR	SSIM
MLEM (B)	9.3822	0.4001
TV (C)	13.4989	0.6341
Proposed 1 (D1)	14.6675	0.6761
Proposed 2 (D2)	17.0831	0.7144

**Table 3.** Quantitative evaluation results for the second phantom.

Method	PSNR	SSIM
MLEM (B)	17.2455	0.7067
TV (C)	17.2679	0.7300
Proposed (D)	22.0501	0.8547

## Data Availability

No new data were created or analyzed in this study. Data sharing is not applicable to this article.

## Abbreviations

The following abbreviations are used in this manuscript:

MLEM: maximum-likelihood expectation-maximization
POCS: projection onto convex sets
PSNR: peak signal-to-noise ratio
SSIM: structural similarity
TV: total variation
